# Endovascular Mechanical Thromboembolus Fragmentation in the Treatment of Critical Pulmonary Thromboembolism in Patients with Acute Hemorrhagic Stroke

**DOI:** 10.17691/stm2020.12.1.09

**Published:** 2020

**Authors:** A.M. Homenko, E.A. Kusmenko, V.V. Pichugin, A.P. Medvedev

**Affiliations:** Physician, Department of Radiosurgical Diagnosis and Treatment, N.A. Semashko Nizhny Novgorod Regional Clinical Hospital, 190 Rodionova St., Nizhny Novgorod, 603126, Russia; Head of the Department of Radiosurgical Diagnosis and Treatment, N.A. Semashko Nizhny Novgorod Regional Clinical Hospital, 190 Rodionova St., Nizhny Novgorod, 603126, Russia; Professor, Department of Anesthesiology, Resuscitation and Emergency Medical Aid, Privolzhsky Research Medical University, 10/1 Minin and Pozharsky Square, Nizhny Novgorod, 603005, Russia; Professor, Department of Hospital Surgery named after B.A. Korolyov, Privolzhsky Research Medical University, 10/1 Minin and Pozharsky Square, Nizhny Novgorod, 603005, Russia

**Keywords:** endovascular mechanical thromboembolus fragmentation, critical PTE, hemorrhagic stroke

## Abstract

**The aim of the study** was to evaluate the effectiveness of endovascular mechanical thromboembolus fragmentation in the treatment of critical pulmonary thromboembolism in patients with acute hemorrhagic stroke.

**Materials and Methods:**

The study included 47 patients with acute hemorrhagic stroke complicated by massive high-risk pulmonary thromboembolism with critical manifestations of the right ventricular failure. All patients were divided into two groups depending on the treatment method: the examined group (n=17) undergone endovascular mechanical thromboembolus fragmentation and the control group (n=30) received only basic intensive therapy.

**Results:**

Thromboembolus fragmentation was performed on patients of the examined group in order to transfer embolism of the trunk and main branches to the smaller branches of the pulmonary artery. The technical success of the procedure (destruction of the central thromboembolus) was achieved in 100% of cases. 14 patients (82.4%) showed positive clinical dynamics: improvement of general condition, reduction of pulmonary artery pressure, decreased volume of pulmonary bed damage according to CT angiography. Three patients (17.6%) died in the early postoperative period. Twenty five patients from the control group died, hospital mortality rate was 83.3%. There were no deaths in the examined group after 6–9 months of follow-up, signs of pulmonary hypertension persisted in 11 patients (64.7%).

**Conclusion:**

Endovascular mechanical thromboembolus fragmentation in the treatment of critical pulmonary thromboembolism in patients with acute hemorrhagic stroke results in fast and safe decrease in pulmonary artery pressure. Fragmentation of central thromboembolus and its displacement into the peripheral vascular bed with a modified pigtail catheter is a minimally invasive surgical procedure which may be used as an alternative to surgical embolectomy in cases of an extremely high risk of surgery and absolute contraindications to thrombolytic therapy.

## Introduction

Hemorrhagic stroke is diagnosed in 1–2 million people each year [1–3]. The survived patients are most vulnerable to venous thromboembolic complications [2–5]. The pulmonary thromboembolism (PTE) rate in this group is less frequent compared with oncological patients and after complicated orthopedic interventions [6]. According to the data of different authors [3, 7, 8], the course of hemorrhagic stroke is complicated by nonfatal PTE in 50% of cases whereas fatal in 1.7–5% of cases.

A great number of works have been devoted to the prevention of PTE in patients with hemorrhagic stroke, however, the problem of treatment of this dreadful disease is far from being solved [4]. Acute pulmonary thromboembolism represents a depressed condition of the cardiovascular system caused by the obstruction of the pulmonary artery bed which is accompanied by potentially reversible right ventricular failure [1]. As a rule, it occurs in patients with massive obstruction of the pulmonary artery bed. The probability of preserving life of these patients depends on rapid and early restoration of the pulmonary arteries blood flow.

Presently, there are various techniques of restoring pulmonary arteries blood flow as a source of treatment: firstly, it is thrombolytic therapy, the golden standard of reperfusion; secondly, surgical embolectomy from the pulmonary arteries; thirdly, endovascular treatment methods [9–11]. In patients with hemorrhagic stroke, it is impossible to use routine thrombolytic therapy due to absolute contraindications and these patients have a very high risk of surgical intervention with cardiopulmonary bypass, so the usage of endovascular method of treatment as a less invasive and traumatic procedure is more preferable.

Catheter fragmentation of thromboemboli is the simplest and most effective method of minimally invasive restoring of the pulmonary arteries blood flow. Displacement of thrombi from the main pulmonary arteries to the lobar and segmental branches reduces the dangerous right ventricle afterload in the majority of cases, and it is the life-saving procedure in patients with acute hemorrhagic stroke (AHS) in a critical state [12].

**The aim of the investigation** was to evaluate the effectiveness of endovascular mechanical thromboembolus fragmentation in the treatment of critical pulmonary thromboembolism in patients with acute hemorrhagic stroke.

## Materials and Methods

The study included 47 patients with AHS complicated with massive high risk PTE with critical manifestations of right ventricular failure. All patients were divided into 2 groups depending on the treatment method. Patients from the examined group (17 patients aged 43–72 years) underwent endovascular mechanical thromboembolus fragmentation. The control group (30 patients aged 50–78 years) received only basic therapy with administration of fractionated/unfractionated heparins for passive thrombus resolution or its size decrease and prevention of fibrin conglomerate deposition on it.

The study was conducted in compliance with the Declaration of Helsinki (2013) and following the approval of the Ethical Committee of Privolzhsky Research Medical University. Written informed consent was obtained from every patient.

To be included in the study, patients should meet the following criteria: acute hemorrhagic stroke, high-risk (PESI>125) critical PTE; embolism of the trunk and main pulmonary arteries; acute right ventricular failure; the possibility to use other methods of pulmonary arterial bed reperfusion (thrombolytic therapy, open pulmonary embolectomy).

Clinical and functional indices are represented in the Table.

**Table:** Clinical presentation of the patients (M±SD)

Parameters	Groups	
	Examined (n=17)	Control (n=30)
Gender (m/f)	7/10	13/17
Age (years)	57.95±8.0	63.20±7.87
Severity of PTE according to PESI (points)	Class V 152±22	Class V 150±24
Operative risk according to ASA physical status classification	Class V	Class V
Initial SPAP (mm Hg)	70.53±4.53	69.20±9.46

Here: SPAP is systolic pulmonary artery pressure.

The decision on advisability of endovascular intervention was made by the council of physicians including the specialists in neurology, cardiology, interventional cardiology, and anesthesiology.

**Figure 1:**
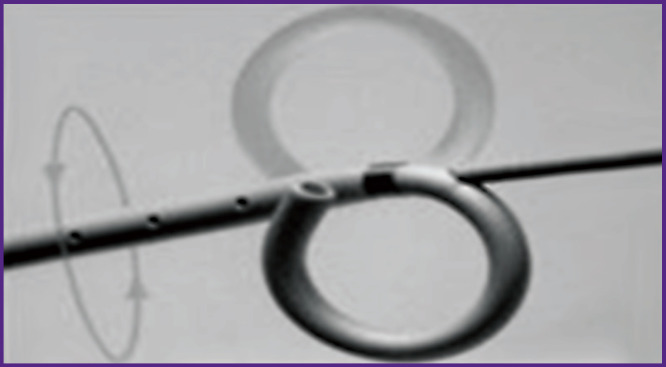
Rotary pigtail catheter for fragmentation of thromboemboli in pulmonary arteries

Thromboembolus fragmentation with a modified pigtail catheter was performed to all patients of the examined group in order to translate embolism of the trunk and main branches into the smaller pulmonary artery branches with subsequent angiopulmonographic control. The treatment methodology consists in selective catheterization of pulmonary arteries using the right-sided jugular or subclavian approach. An angiographic guide wire is inserted into the thrombus mass and then a modified pigtail catheter (5–6 F) (Figure 1) is advanced along it and positioned in the pulmonary artery [13]. The angiographic guide wire in the distal catheter goes out through the side rather than the end openings. The coiled inner end of the catheter placed near the guide wire fragments the thromboembolus by rotating movements.

Patients underwent ultrasound investigation 24 h after the procedure, and on day 7, angiographic control (CT) was performed. The efficacy of the procedure was assessed by the data of all investigations and clinical features.

The data were statistically processed using Microsoft Office Excel program. Categorical variables were expressed in percentage terms; constant variables were presented as М±SD where M is mean, SD standard deviation. The main characteristics and hospital outcomes for the two groups were evaluated using Student’s t-test.

## Results

All patients were in class V according to ASA classification. It means that these patients may be expected to die within 24 h whether the operative intervention will be performed or not. Class V according to PESI classification (130–174 points) designates an extremely high risk of death (10–24%).

**Figure 2:**
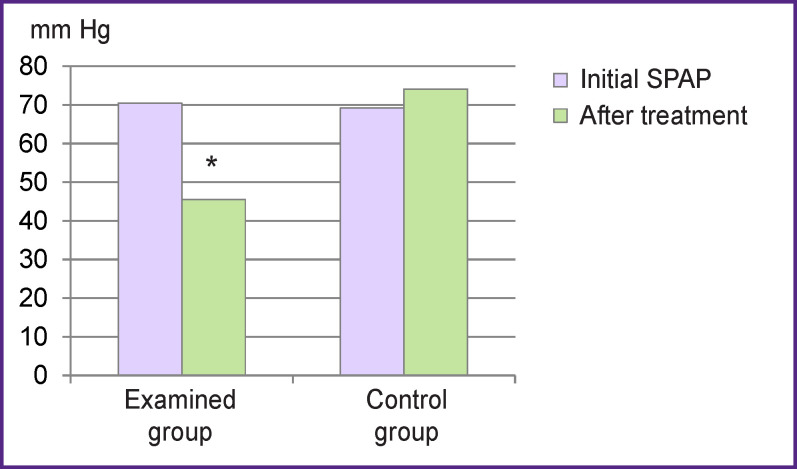
Dynamics of systolic pulmonary artery pressure (SPAP) before and after treatment; * p≤0.05

Thromboembolus fragmentation was performed to the patients of the examined group. The technical success of the procedure (destruction of the central thromboembolus) has been achieved in 100% of cases. 14 patients (82.4%) showed positive clinical dynamics: improvement of general condition, reduction of pulmonary artery pressure, and decrease in the obstructed pulmonary bed square confirmed by CT angiography. Changes in systolic pulmonary artery pressure before and after the endovascular intervention are presented in Figure 2. Three patients (17.6%) died in the early postoperative period due to the initial severity of their state. 25 patients of the control group (from 30 patients) died, hospital mortality rate was 83.3%.

There were no deaths in the examined group in the remote period (6–9 months later), ultrasound control showed persistent signs of pulmonary hypertension in 11 patients (64.7%).

A case of critical PTE successful treatment in woman with AHS using endovascular destruction of thromboemboli in the main branches of the pulmonary artery is presented below.

*Patient N., 56 years old, admitted to the Neurological Intensive Care Unit with acute hemorrhagic stroke in the right mesencephalic artery area. A sharp worsening of her state occurred 8 days after the hemorrhagic event and her condition was evaluated as extremely severe. The level of consciousness impairment according to Glasgow Coma Scale was 13 points (deafening); there was also marked hypoxemia SatO_2_=84% with oxygen inhalation through nasal catheters; arterial hypotension: BP — 90/50 mm Hg requiring the inotropic myocardial stimulation; HR — 110–120 bpm. Obstructed pulmonary arteries blood flow, dilation of the right heart chambers and the left atrium were found by the ultrasound investigation. Left ventricle hypertrophy, mitral valve insufficiency with moderate regurgitation (grade II), tricuspid valve regurgitation (grade III), signs of severe pulmonary hypertension, and hydropericardium were registered. Left ventricular ejection fraction was 55%, systolic pulmonary artery pressure — 79 mm Hg.*

**Figure 3:**
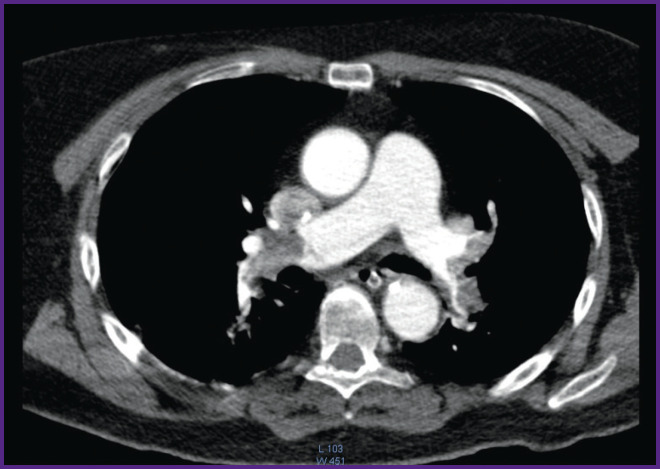
Patient N., 56 years old. MSCT angiography of the pulmonary arteries. Presence of thromboemboli in the distal parts of the pulmonary arteries with obstruction of their lumen

*MSCT with angiography of the pulmonary arteries revealed the presence of thrombi in the distal parts of the pulmonary artery occluding their lumens (Figure 3).*

**Figure 4:**
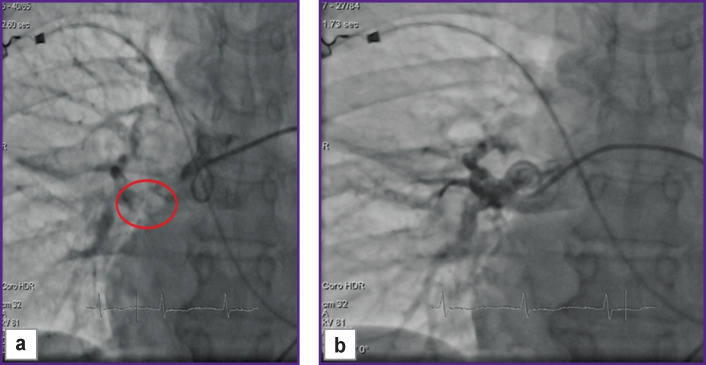
Insertion of the rotary pigtail catheter (а) and thromboembolus fragmentation in the pulmonary arteries under control (b)

*The council of physicians consisting of the anesthesiologist, cardiologist, cardiovascular surgeon, neurologist, and interventional cardiologist was conducted. The patient’s state was assessed as critical (class V ASA) complicated by massive PTE. Patient had an absolute contraindication to thrombolytic therapy due to acute hemorrhagic stroke. Surgical thromboembolectomy could not be indicated as a high operative risk procedure. By life-saving indications, catheter destruction of thromboemboli in the main branches of the pulmonary artery was performed. Thromboemboli were mechanically fragmented by a pigtail catheter (F6) (Figure 4).*

*After the procedure, the patient’s state improved: systolic pulmonary artery pressure decreased from 79 to 57 mm Hg, SatO_2_ increased up to 92–94%, left ventricular ejection fraction increased to 65%. The postoperative period was uneventful.*

## Discussion

Patients with PTE are a heterogeneous group differing in gender, age, etiology and location of thromboemboli, severity of pulmonary blood flow disturbances and central hemodynamics, clinical manifestations of the disease, previous cardiopulmonary status, concurrent illnesses aggravating their state and limiting and even hindering the use of the necessary medical procedures. Methods and drugs for PTE treatment are aggressive, not always give the required clinical effect, may be accompanied by various complications and side-effects, therefore, their application requires cogent argumentation.

A key to PTE pathogenesis is the thromboembolic obstruction of pulmonary arteries. In this regard, the current strategy of patient management demands urgent decision-making on thromboembolus localization and the method of blood flow restoration in the pulmonary artery.

Thrombolytic therapy and surgical intervention are considered as the most effective ways to restore pulmonary artery patency. In recent years, the role and place of PTE surgical treatment have changed substantially owing to the technological advances, gained experience of surgeons, and improvement of their skills [10–12]. The scope of indications to surgery has broadened: thromboembolism of the pulmonary artery trunk or its main branches in case of shock, acute right ventricular failure, low level of arterial oxygen saturation, or contraindications to thrombolytic therapy.

A great number of scientific works devoted to various aspects of PTE prevention, diagnosis and treatment have been published [2, 3, 9–12]. However, unsolved remain problems of treating thromboembolism in patients with hemorrhagic stroke in a critical state. The obtained results allow us to consider that catheter fragmentation of thromboemboli is the simplest and most effective minimally invasive method of pulmonary artery flow restoration.

According to the data of European Society of Cardiology, catheter thromboembolus fragmentation is not less effective than surgical embolectomy from pulmonary arteries. Moreover, this method may be used in patients whose condition excludes traditional operative intervention due to a high risk, while thrombolytic means can result in hemorrhagic complications [12].

Karpenko et al. [14] have shown a high effectiveness of catheter thromboembolus fragmentation in combination with local thrombolysis. They describe treatment of 164 patients with PTE. Complete or substantial lysis of thromboemboli with normalization of the systemic hemodynamics was achieved in 103 (63%) cases. Partial thromboemboli lysis with stabilization of systemic hemodynamics was noted in 52 (32%) patients. No clinical improvement was observed in 9 patients.

According to other authors, catheter thromboembolus fragmentation was performed in 20 patients with massive PTE (the obstructed pulmonary arterial bed occupied over 50%). Fragmentation of thromboemboli was supplemented by administration of thrombolytic drugs in 17 patients. Three patients had absolute contraindication to thrombolytic therapy due to severe central nervous system injury (traumatic brain injury, brain tumor, neurosurgery). Two of them survived. The total mortality rate was 20% (4 of 20 patients).

All our patients (17 patients with hemorrhagic stroke) had absolute contraindications to thrombolytic therapy and this method could not be employed. Three patients died in the nearest postoperative period (mortality rate — 17.65%).

## Conclusion

Endovascular mechanical thromboembolus fragmentation in the treatment of critical pulmonary thromboembolism in patients with acute hemorrhagic stroke results in fast and safe decrease in pulmonary artery pressure. Fragmentation of central thromboembolus and its displacement into the peripheral vascular bed with a modified pigtail catheter is a minimally invasive surgical procedure which may be used as an alternative to surgical embolectomy in cases of an extremely high risk of surgery and absolute contraindications to thrombolytic therapy.
